# Radiodiagnostics of standard orthodontic radiographs—dental and extradental incidental findings: Do we see everything we should see?

**DOI:** 10.1007/s00056-023-00483-1

**Published:** 2023-07-04

**Authors:** Bernhard Wiechens, Daniela Klenke, Anja Quast, Petra Santander, Ida Skorna, Philipp Meyer-Marcotty

**Affiliations:** Department of Orthodontics, University Medical Center Goettingen, Robert-Koch-Str. 40, 37075 Goettingen, Germany

**Keywords:** Orthopantomography, Lateral cephalogram, Incidental discovery, Surveys and questionnaires, Craniofacial abnormalities, Orthopantomographie, Fernröntgenseitenaufnahme, Zufallsbefund, Umfragen und Fragebögen, Kraniofaziale Anomalitäten

## Abstract

**Objectives:**

The extent of undetected incidental findings in routine orthodontic radiographs is still unknown. However, incidental findings that are not in the primary focus of orthodontic diagnostics may be of high medical relevance. Therefore, this study aimed to analyse whether incidental findings are reliably detected and which parameters influence the orthodontist’s assessment.

**Methods:**

In a clinical cross-sectional study 134 orthodontists evaluated two orthopantomogram (OPT) and two lateral cephalogram (LC) radiographs each via a standardised online survey. The radiographs were previously examined by three dentists and one radiologist—in a pilot phase—regarding the number of incidental findings and subsequently defining as gold standard in a consensus procedure. The radiographs were presented consecutively, the number of incidental findings detected were noted and the individual findings could be described in free text form.

**Results:**

Overall, 39.1% of the incidental findings were detected. The orthodontists’ focus was primarily on the dental region. Here, 57.9% of incidental findings were detected, while 20.3% were detected in extradental regions (*p* < 0.001). A highly relevant finding of suspected arteriosclerotic plaque was detected in 7.5% of cases (OPT). Significantly more incidental findings were detected on OPTs than on LCs (OPT 42.1%, LC 36.0%, *p* < 0.001). As participants’ length of professional experience increased, significantly more time was spent on the assessment (*p* < 0.001), correlating positively with the detection of incidental findings.

**Conclusions:**

Even in daily routine practice, attention must be paid to a thorough assessment of all radiographed regions. The factors time and professional experience can prevent practitioners from overlooking findings outside the orthodontic focus.

## Abstract (de)

**Zielsetzung:**

Das Ausmaß unentdeckter Zufallsbefunde in kieferorthopädischen Routineröntgenaufnahmen ist noch immer unbekannt. Zufallsbefunde, die nicht im primären Fokus der kieferorthopädischen Diagnostik stehen, können eine hohe medizinische Relevanz haben. Ziel dieser Studie war es daher zu analysieren, ob Zufallsbefunde zuverlässig erkannt werden und welche Parameter die Beurteilung durch die Kieferorthopäd*innen beeinflussen.

**Methoden:**

In einer klinischen Querschnittsstudie bewerteten 134 Kieferorthopäd*innen je 2 Orthopantomogramme (OPT) und 2 Fernröntgenseitenaufnahmen (FRS) mittels standardisierter Online-Umfrage. Im Rahmen einer Pilotphase waren die Röntgenbilder zuvor von 3 Zahnärzten und einem Radiologen auf die Anzahl der Zufallsbefunde geprüft worden und anschließend in einem Konsensverfahren als Goldstandard definiert. Die Röntgenbilder wurden fortlaufend präsentiert, die Anzahl der entdeckten Zufallsbefunde notiert und die einzelnen Befunde in Freitextform beschrieben.

**Ergebnisse:**

Insgesamt wurden 39,1 % der Zufallsbefunde entdeckt. Der Fokus der Kieferorthopäd*innen lag vor allem auf dentaler Ebene. Hier wurden 57,9 % der Zufallsbefunde entdeckt, während 20,3 % in extradentalen Regionen entdeckt wurden (*p* < 0,001). Ein hochrelevanter Befund mit Verdacht auf arteriosklerotische Plaque wurde in 7,5 % der Fälle festgestellt (OPT). Bei OPTs wurden signifikant mehr Zufallsbefunde entdeckt als bei FRS-Aufnahmen (OPT: 42,1 %, FRS: 36,0 %, *p* < 0,001). Mit zunehmender Berufserfahrung der Teilnehmer*innen wurde signifikant mehr Zeit für die Beurteilung aufgewendet (*p* < 0,001), was positiv mit der Erhebung von Zufallsbefunden korrelierte.

**Schlussfolgerungen:**

Auch in der alltäglichen Praxis muss auf eine gründliche Beurteilung aller röntgenologisch erfassten Regionen geachtet werden. Die Faktoren Zeit und Berufserfahrung können den Behandler davor bewahren, Befunde außerhalb des kieferorthopädischen Fokus zu übersehen.

## Introduction

Lateral cephalograms (LC) and orthopantomograms (OPT) are taken and analysed daily in orthodontic practices for diagnostics and treatment planning. Approximately 725,800 LCs and 1,191,900 OPTs were taken in the context of orthodontic queries in Germany in 2019 [18]. It is an obligatory process for each orthodontist to carry out thorough assessment and documentation of the entire X‑ray. They are the experts who are indicating, performing and diagnosing the diagnostic examinations. Both OPT and LC result in a field of view in which a large area of the entire craniofacial complex is displayed. Besides the dental region, numerous structures of the facial and cervical region are depicted, which considerably expand the scope of findings in orthodontics [27]. Simultaneously, incidental findings often occur outside the dental region [21]. Additional expertise related to this specific anatomical region and accurate radiographic assessment of the area needs to be reinforced through specialised continued medical education in orthodontics.

Practitioners are aware that they are likely to encounter incidental findings in the orthodontic evaluation of OPTs and/or LCs. Nevertheless, there are surprisingly few controlled studies on this topic [16]. The majority of literature related to this topic derives from primarily descriptive studies of individual findings [11, 15, 26, 32, 35–37], where the reported prevalence within the scope of orthodontic diagnostics vary significantly: Bondemark et al. [9] identified pathological findings or anomalies on OPTs in barely 9% of orthodontic patients, whereas Hernandez et al. [16] detected incidental findings in addition to the primary focus of the radiographic indication in about 88% of the examined OPTs and LCs.

The clinical importance of this topic is further highlighted by the fact that 50% of orthodontists encounter at least once in their professional life an incidental finding on an LC involving a potentially life-threatening pathology [23]. Furthermore, pathological incidental findings can be of considerable importance for the orthodontic treatment course and may result in a therapeutic modification [1, 19, 22]. Vice versa, irrelevant incidental findings have no impact for the patient and therefore do not require further radiographic monitoring or additional diagnostic examinations. Against this clinical background, the present study analysed the assessment quality of OPT and LC images in orthodontic practices with the following questions:

- How frequently are incidental findings detected during standard orthodontic X‑ray examinations?
- Is there a difference in regard to the prevalence of incidental findings between dental and extradental regions on OPTs and LCs?
- Are there examiner-independent parameters that affect the reliable detection of incidental findings?

## Methods

The clinical observational study with a cross-sectional design was conducted at the Department of Orthodontics of the University Medical Center Goettingen. Data acquisition was performed between 17 July and 21 October 2020. Approval was granted by the ethics committee of the University Medical Center Goettingen (ethics number 16/11/19). The study was conducted in accordance with the Declaration of Helsinki. The report of the study complied with the Strengthening the Reporting of Observational Studies in Epidemiology (STROBE) guidelines in its entirety. The sample size calculation was performed using G*Power 3.1.6 (University of Düsseldorf; Germany) [12] and R (version 3.6.3; The R Foundation for Statistical Computing; Vienna; Austria) [29] and yielded a minimum sample size of 89 participants.

### Questionnaire design—sociodemographic data

An online navigated questionnaire (LimeSurvey GmbH, Hamburg, Germany) was used to collect data on the practical assessment of orthodontic radiographs by dentists, focusing on the number and location of incidental findings to be identified.

The radiographs used for the study were analysed in a first phase with a multistage calibration procedure and determined to be the gold standard for the present study [21]:

An experienced radiologist, an orthodontist, an oral surgeon and a dentist assessed a total of 300 OPT images and 300 LC images (Orthophos XG Plus DS/Ceph 9200, Sirona Dental Systems GmbH, Bensheim, Germany) [21]. In addition to the valid indication that justified radiation exposure (primary finding), additional secondary findings were analysed in this pilot study with regard to their number, location and relevance.

**Fig. 1:**
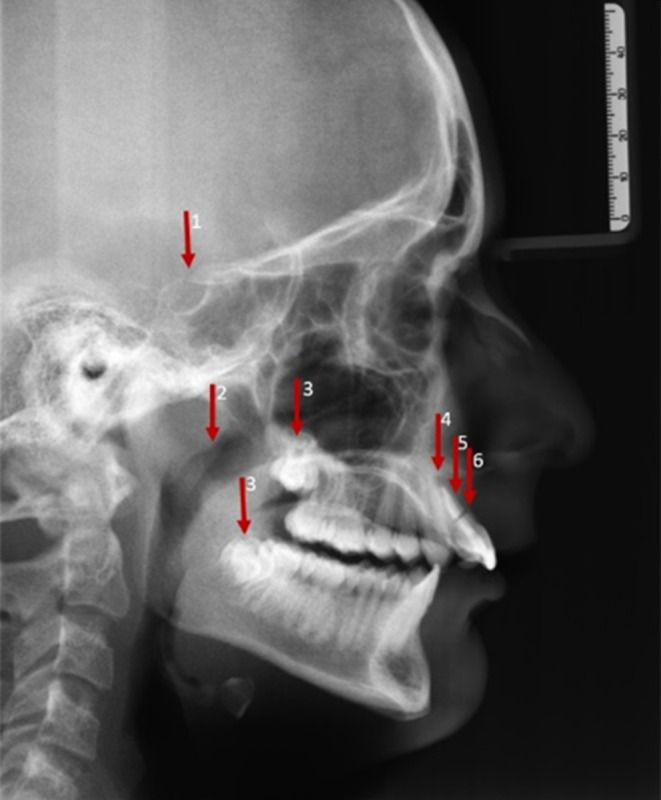
Lateral cephalogram image of an adolescent patient with six incidental findings. Extradental incidental findings: tendency sella bridging (1), hypertrophic adenoids (2), apical radiolucency UJ incisor (4). Dental incidental findings: impacted third molar UJ/LJ (3), endodontic filling UJ incisor (5), fracture UJ incisor (6). *UJ* upper jaw, *LJ* lower jaw Fernröntgenseitenaufnahme eines jugendlichen Patienten mit 6 Zufallsbefunden. Extradentale Zufallsbefunde: Tendenz zur Sella-Brücke (1), hypertrophe Adenoide (2), apikale Radioluzenz OK-Inzisivus (4). Zahnärztliche Zufallsbefunde: Impaktierter dritter Molar OK/UK (3), endodontische Füllung OK-Inzisivus (5), Fraktur OK-Inzisivus (6). *OK *Oberkiefer, *UK *Unterkiefer

**Fig. 2:**
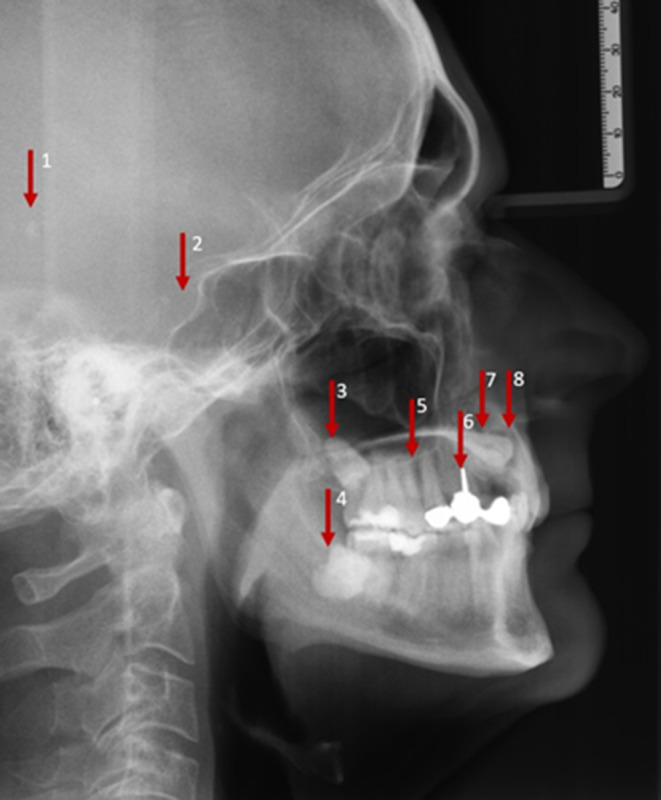
Lateral cephalogram image of an adult patient with eight incidental findings. Extradental incidental findings: opacity of posterior cranial fossa (1), atypical sella morphology (2), deep recess of maxillary sinus (5). Dental incidental findings: impacted third molar UJ/LJ (3), radiopaque structure 48 (4), endodontic filling UJ premolar (6), displaced canine UJ (7), root resorption UJ anterior (8). *UJ* upper jaw, *LJ* lower jaw Fernröntgenseitenaufnahme eines erwachsenen Patienten mit 8 Nebenbefunden. Extradentale Zufallsbefunde: Trübung der hinteren Schädelgrube (1), atypische Morphologie der Sella (2), tiefer Recessus der Kieferhöhle (5). Dentale Zufallsbefunde: impaktierter dritter Molar OK/UK (3), röntgenopake Struktur 48 (4), endodontische Füllung OK Prämolar (6), verlagerter Eckzahn OK (7), Wurzelresorption OK anterior (8). *OK *Oberkiefer, *UK *Unterkiefer

**Fig. 3:**
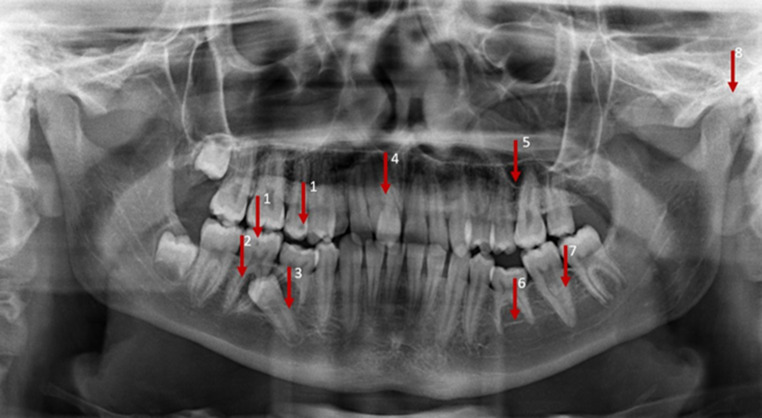
Orthopantomogram image of an adolescent patient with eight incidental findings. Extradental incidental findings: suspected cyst 45 (2), deep recess of maxillary sinus (5), temporomandibular joint abnormality (8). Dental incidental findings: caries 15 and/or 46 (1), displaced 45 (3), atypical eruption region 11–13 (4), agenesis 35 (6), conical root 36 (7) Orthopantomogramm-Aufnahme eines jugendlichen Patienten mit 8 Nebenbefunden. Extradentale Nebenbefunde: Verdacht auf Zyste 45 (2), tiefer Recessus der Kieferhöhle (5), Kiefergelenkanomalie (8). Dentale Zufallsbefunde: Karies 15 und/oder 46 (1), verlagerter 45 (3), atypische Eruptionsregion 11–13 (4), Agenesie 35 (6), konische Wurzel 36 (7)

**Fig. 4:**
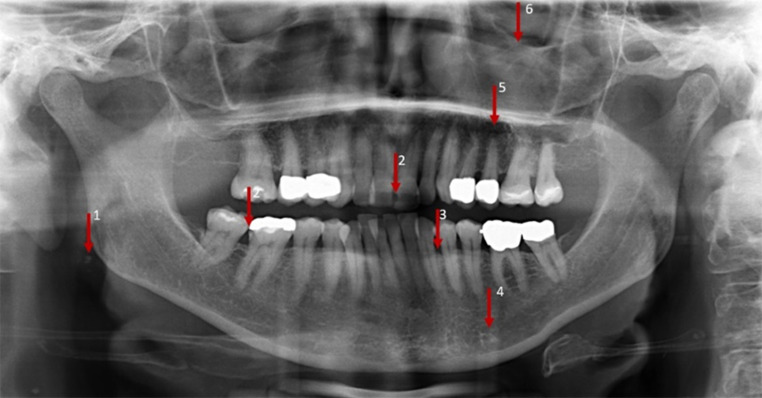
Orthopantomogram image of an adult patient with six incidental findings. Extradental incidental findings: arteriosclerosis of the external carotid artery (1), suspected periodontitis UJ/LJ (3), sclerosis of the left anterior mandible (mandibular region located apical of lower second premolar) (4), suspected apical radiolucency 25 (5), maxillary sinus opacity (6). Dental incidental findings: caries 46 and/or 21 (2). *UJ* upper jaw, *LJ* lower jaw Orthopantomogramm-Aufnahme eines erwachsenen Patienten mit 6 Nebenbefunden. Extradentale Zufallsbefunde: Arteriosklerose der Arteria carotis externa (1), Verdacht auf Parodontitis OK/UK (3), Sklerose des linken vorderen Unterkiefers (Unterkieferregion apikal des unteren zweiten Prämolaren) (4), Verdacht auf apikale Radioluzenz 25 (5), Opazität der Kieferhöhle (6). Dentale Zufallsbefunde: Karies 46 und/oder 21 (2). *OK *Oberkiefer, *UK *Unterkiefer

Based on these data, one OPT and LC radiograph were selected from adolescent (OPT: female, 15.6 years, LC: male, 13.5 years) and adult patients (OPT: female, 72.9 years, LC: male, 52.8 years) for the present study (Figs. 1, 2, 3 and 4). Inclusion criteria were the similar distribution of the total number of incidental findings as well as the subdivision into “extradental” and “dental” incidental findings in adolescents and adults. This ensured the comparability of the categories for quantitative analysis.

The online questionnaire was divided into three sections to ensure standardisation for all investigators participating in the survey. In the first section, participants were given important information about the survey design and calibration by the examiners using a sample radiograph (OPT) with sample findings. The explanations included the instruction to examine 4 radiographs (2 OPTs, 2 LCs), to indicate detected incidental findings separated by commas in the answer field, and to indicate a classification directly after the naming of the finding as (A) no further diagnosis or therapy needed or (B) unclear finding, further diagnosis or therapy needed. The definition for an incidental finding was “finding that cannot be detected during the clinical examination of the patient and was not the indication for radiography”. The additional classification differentiated A (no further diagnosis or therapy needed) from B (unclear finding, further diagnosis or therapy needed). For reminding purposes, the definition of findings and classifications was displayed in blue font below the radiographs over the entire scope of the survey. Finally, a generally brief, simple statement of findings was requested, with separation by commas in the case of multiple incidental findings. The second section collected sociodemographic details of the examiners concerning professional experience (indication from < 5 to > 25 years in 5‑year increments) and sex (male, female, not applicable). In the third section, the actual evaluation of the four radiographs was performed starting with the adolescent and adult LC, followed by the adolescent and adult OPT, using the previously described procedure.

### Method and conduct of the survey

Initially, a pilot phase took place in the Department of Orthodontics, University Medical Centre, Göttingen, in which ten orthodontists assessed the radiographs implemented in LimeSurvey (LimeSurvey GmbH, Hamburg, Germany). After this pilot phase had been evaluated, the online questionnaire for the main survey was adapted and a total of four X‑rays were selected. The survey was sent to freely and publicly accessible email addresses of orthodontists in practices and university departments across Germany. A total of 2615 potential participants from orthodontic treatment practices who provided a free and publicly accessible email address were included. The survey was sent out to all 2615 email addresses on 17 July 2020. Four weeks later, on 14 August 2020, the first reminder was sent to everyone who had not yet taken part in the survey up to that date. Another four weeks later, on 16 September 2020, the second and final reminder was dispatched. The survey remained active for another 5 weeks after the final reminder—hence for a total of 13 weeks. Participants received a personalised link which only allowed participation itself to be tracked, thus ruling out the possibility of examiners participating several times. Owing to the study design as an online survey, the examination conditions were not the same for every participant. As every orthodontist usually has a diagnostic monitor available, the study format assumed that the radiographs were also diagnosed on this monitor. However, this cannot be clearly assured due to the nature of the online design. The participants’ answers were pseudonymised—therefore, it was not possible to track which participant had given which answers. After participation, it was no longer possible to retract an answer because, from that point onwards, it was no longer possible to assign a particular answer to a participant.

### Statistical analysis

Statistical analysis of the results was performed using Excel® (Microsoft Corporation, Redmond, WA, USA) and SPSS® (Statistical Package for Social Science®, version 27, IBM, Armonk, NY, USA). Analysis of the questionnaire results was performed by means of descriptive statistics with the data listed as mean (number of findings) (M), standard deviation (SD), minimum (min) and maximum (max). The inductive statistics in relation to the imaging category (LC vs. OPT; dental findings vs. extradental findings; imaging adolescent–U18 [under 18 years of age] vs. imaging adult–O18 [older than 18 years of age]; and A finding vs. B finding) were performed using t‑tests. Subsequently, associations between the above-named imaging categories and examiner categories (professional experience in years; time—assessment time in seconds) were analysed using Kendall’s tau‑b (τb; not normal distribution) or using an unpaired t‑test (gender).

## Results

### Sociodemographic data

Of the 2615 participants contacted, the assessment questionnaires of 422 participants could be collected, which represented a 16.1% raw response rate. A total of 138 questionnaires were returned completely, four responses were removed from the analysis because the participants did not meet the inclusion criteria. In the final analysis, the complete data of 134 participants in total could be analysed in its entirety. Their mean age was 45.7 ± 11.0 years (minimum 25 years; maximum 70 years). This resulted in a total response rate of 5.3% for the survey. The respondents comprised 57 men (42.5%), 76 women (56.7%) and one unspecified participant (0.7%).

### Results analysis

The number of incidental findings detected was categorised as total incidental findings, incidental findings on OPTs and LCs, incidental dental and extradental findings, findings in adolescent patients (U18) and adults (O18), and category A and B (Table 1). On average, the participants detected 10.9 ± 3.1 (39.1%) of the 28 possible incidental findings summed from all four radiographs. 5.0 ± 1.6 (36.0%) of the 14 possible incidental findings were detected on LCs, while 5.9 ± 2.0 (42.1%) were identified on OPTs. When comparing the two imaging techniques, incidental findings were identified significantly more frequently on OPTs than on LCs (*p* < 0.001).

**Table 1:** Overview of the number of incidental findings to be detected Übersicht über die Anzahl der zu entdeckenden Zufallsbefunde

	*n* (100%)	Min (in %)	Max (in %)	M ± SD (in %)	*p*-value
Incidental findings total	28	4 (14.3)	21 (75.0)	10.9 ± 3.1 (39.1)	–
LC	14	2 (14.3)	11 (78.6)	5.0 ± 1.6 (36.0)	< 0.001***
OPT	14	2 (14.3)	12 (85.7)	5.9 ± 2.0 (42.1)	
Dental incidental findings	14	2 (14.3)	14 (100)	8.1 ± 2.1 (57.9)	< 0.001***
Extradental incidental findings	14	0 (0)	9 (64.3)	2.8 ± 1.8 (20.3)	
Adolescent (U18)	14	2 (14.3)	12 (85.7)	6.1 ± 2.0 (43.4)	< 0.001***
Adult (O18)	14	1 (7.1)	10 (71.4)	4.9 ± 1.6 (34.8)	
A findings	7	0 (0)	6 (85)	2.9 ± 1.4 (41.3)	0.074
B findings	21	3 (14.3)	15 (71.4)	8.1 ± 2.4 (38.3)	

*n* number of possible findings,* Min* Minimum number of findings, *Max* maximum number of findings, *M* mean, *SD* standard deviation values of the detected findings are grouped by imaging type (*OPT* orthopantomogram vs *LC* lateral cephalogram), location (dental/extradental), *U18* adolescent, *O18* adult; finding classification: *A findings *no further diagnosis or therapy needed vs. *B findings * unclear finding, further diagnosis or therapy needed

***p < 0.001

Regarding the location, 8.1 ± 2.1 (57.9%) of the possible 14 incidental findings were identified in the dental area; in contrast, significantly fewer incidental findings were detected in the extradental area with only 2.8 ± 1.8 (20.3%) (*p* < 0.001). Regarding imaging in adolescents (U18) versus imaging in adults (O18), the analysis revealed that significantly more incidental findings were detected in adolescents (U18) with an average of 6.1 ± 2.0 (43.4%) than in adults (O18) with 4.9 ± 1.6 (34.8%; *p* < 0.001). Comparison of detected incidental findings by classification revealed no significant difference (A findings 41.3%, B findings 38.3%, *p* = 0.074).

Table 2 provides a detailed listing of all incidental findings by detection (findings identified by the examiners), location (dental/extradental) and classification (A/B findings) according to the type of the radiograph (LC/OPT). The findings on LCs which were detected by the majority of the participants were a fracture of an incisor (94.0%; LC 1), a displaced canine (96.3%; LC 2) and impacted third molars (86.6%; LC 2). Hypertrophic adenoids (epipharynx), a deep recess of the maxillary sinus (LC 1 or LC 2; 11.9% in each case), a tendency to sella bridging (anterior cranial base, LC 1; 9.7%) and an opacity in the posterior cranial fossa (posterior cranial base, LC 2; 0.7%) were noted by only a few examiners. On OPTs, a displaced tooth 45 (OPT 1; 90.3%), agenesis of the lower second premolar (OPT 1; 84.3%) and an atypical eruption in region 11–13 (OPT 1; 82.8%) were detected by most of the participants, whereas the findings of an apical radiolucency at 25 (OPT 2; 11.2%), arteriosclerosis of the external carotid artery (OPT 2; 7.5%) and sclerosis of the left anterior mandible (mandibular region located apical of lower second premolar) (OPT 2; 0%) were rarely or not at all detected. A descriptive analysis of the location of findings demonstrated that incidental findings were more frequently identified in the dental region, whereas rarely detected incidental findings were primarily located extradentally.

**Table 2:** Detailed list of all the findings^a^ to be detected in three categories Detaillierte Auflistung aller zu erkennenden Befunde^a^ in 3 Kategorien

Radiograph	Incidental finding	Location (dental/extradental)	Class (A/B)	Detected findings *n* (%)
LC 1 (adolescent)	Fracture UJ incisor	Dental	B	126 (94.0)
	Endodontic filling UJ incisor	Dental	A	48 (35.8)
	Apical radiolucency UJ incisor	Extradental	B	26 (19.4)
	Tendency sella bridging	Extradental	B	13 (9.7)
	Hypertrophic adenoids	Extradental	B	16 (11.9)
	Impacted third molar UJ/LJ	Dental	B	51 (38.1)
LC 2 (adult)	Displaced canine UJ	Dental	B	129 (96.3)
	Impacted third molar UJ/LJ	Dental	B	116 (86.6)
	Deep recess of maxillary sinus	Extradental	A	16 (11.9)
	Opacity posterior cranial fossa	Extradental	B	1 (0.7)
	Radiopaque structure 48	Dental	B	31 (23.1)
	Atypical sella morphology	Extradental	B	32 (23.9)
	Root resorption UJ anterior	Dental	B	34 (25.4)
	Endodontic filling UJ premolar	Dental	A	36 (26.9)
OPT 1 (adolescent)	Suspected cyst 45	Extradental	B	39 (29.1)
	Agenesis 35	Dental	A	113 (84.3)
	Displaced 45	Dental	B	121 (90.3)
	Deep recess of maxillary sinus	Extradental	A	40 (29.9)
	Conical root 36	Dental	A	23 (17.2)
	TMJ abnormality	Extradental	B	31 (23.1)
	Atypical eruption region 11–13	Dental	A	111 (82.8)
	Caries 15 and/or 46	Dental	B	55 (41.0)
OPT 2 (adult)	Suspected periodontitis UJ/LJ	Extradental	B	83 (61.9)
	Maxillary sinus opacity	Extradental	B	59 (44.0)
	Caries 46 and/or 21	Dental	B	91 (67.9)
	Suspected apical radiolucency 25	Extradental	B	15 (11.2)
	Sclerosis of the left anterior mandible (mandibular region located apical of lower second premolar)	Extradental	B	0 (0)
	Arteriosclerosis of the external carotid artery	Extradental	B	10 (7.5)

^a^Findings grouped by detection (proportion of findings identified by examiners in absolute values and percent), location (classification by examiners as dental/extradental incidental finding) and classification (class, examiners’ classification as A/B finding)

*LC* Lateral cephalogram, *OPT* Orthopantomogram, *UJ* upper jaw, *LJ* lower jaw, *A findings *no further diagnosis or therapy needed, *B findings* unclear finding, further diagnosis or therapy needed

### Assessment time

**Fig. 5:**
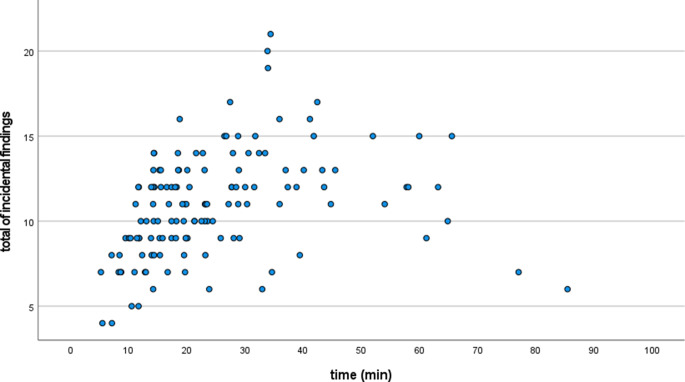
Scatter plot of detected incidental findings against assessment time. Distribution of detected incidental findings as a function of time in absolute values Streudiagramm der entdeckten Zufallsbefunde in Abhängigkeit von der Untersuchungszeit. Verteilung der entdeckten Zufallsbefunde in Abhängigkeit der Zeit in absoluten Werten

The mean assessment time of all the examiners was 25.1 ± 15.0 min. A significant correlation between the time required and the number of incidental findings detected could be demonstrated in both imaging techniques (*p* < 0.001 in each case, Table 3). Thus, more incidental findings overall were detected if the assessment time increased. Analysis of the incidental findings on the two different types of radiographs as well as in the dental and extradental regions also benefited significantly from a longer assessment time. The number of incidental findings detected increased continuously with the examination duration and peaked at 13.8 ± 4.7 incidental findings in the range of 30–35 min. The extension of examination duration beyond 35 min did not lead to a further increase in the number of incidental findings detected (Fig. 5).

**Table 3:** Results of the statistical analysis of associations between the parameters Ergebnisse der statistischen Analyse der Zusammenhänge zwischen den Parametern

Incidental findings^a^	Professional experience (Kendall’s τ)	Assessment time (Kendall’s τ)	Gender (Unpaired t‑test)
Total	0.087	< 0.001***	0.826
LC	0.018*	< 0.001***	0.935
OPT	0.435	< 0.001***	0.695
Dental	0.005**	< 0.001***	0.580
Extradental	0.621	< 0.001***	0.787

^a^Incidental findings detected in total, on lateral cephalogram (LC), orthopantomogram (OPT), as well as dental and extradental incidental findings grouped by professional experience, assessment time and gender

*p < 0.05

**p < 0.01

***p < 0.001

### Professional experience and gender

Professional experience was shown to have an influence on the detection of incidental findings on LC images and in the dental region (Table 3). With increasing professional experience of the examiners, it was found that significantly more incidental findings were detected on LCs (*p* = 0.020) and in dental regions (*p* = 0.008). A summation of the incidental findings overall, as well as an individual analysis with reference to OPTs and the extradental region showed no significant correlation with professional experience. With increasing professional experience, participants took significantly longer to perform the assessment (*p* < 0.001). Table 4 displays the number of incidental findings detected compared to the total number of incidental findings according to professional experience. Participants having less than 5 years of professional experience detected the fewest incidental findings with an average of 9.7 ± 2.8, while participants with 20 years of professional experience detected the most incidental findings with an average of 12.1 ± 2.0 out of a total of 28 on all four radiographs. The participants’ gender had no significant influence on the number of incidental findings detected in any area (Table 3).

**Table 4:** Incidental findings detected as a function of professional experience Entdeckte Zufallsbefunde in Abhängigkeit von der Berufserfahrung

Professional experience years	Participants *n* (%)	Detected incidental findings total M ± SD
Max. 5	25 (18.7)	9.7 ± 2.8
Max. 10	19 (14.2)	11.7 ± 3.0
Max. 15	21 (15.7)	10.1 ± 3.4
Max. 20	20 (14.9)	12.1 ± 2.0
Max. 25	19 (14.2)	10.7 ± 3.6
> 25	30 (22.4)	11.5 ± 3.2

*n* number of participants, *M* *±* *SD* mean ± standard deviation, *Max*. maximum

## Discussion

A thorough, appropriate assessment of radiographs that includes all depicted structures is a prerequisite for accurate diagnosis in high-quality orthodontics. Against this background, the approach of this clinical study was to determine the extent to which orthodontists recognise incidental findings on standardised radiographs (LC/OPT) and whether examiner-dependent or image-dependent parameters influence the quality of their assessment.

For the study, a total of 2615 orthodontists were contacted by email to participate in an online survey. Online surveys are the most economical method of data collection [33] and offer many advantages (wide distribution/accessibility; simple and rapid data collection). However, a generally lower response rate was also reported [20]. At 5.3%, the net response rate in this study can be considered acceptable for online surveys: Obermann et al. [25] reported a net response rate of 4.3% for a survey among general practitioners and described this as a high value for a spontaneous, online-based survey. The rate observed in this study may thus be interpreted as representative for online surveys [25].

The present study minimised the time needed for examination with only four radiographs to be examined. Nevertheless, the average processing time was about 25 min, with large individual variations. This time requirement helps to explain the difference between raw and net response rates mentioned above. The radiographs showed either six or eight incidental findings, and the individual groups were comparable quantitatively. Care was also taken to ensure that an equal total number of incidental dental and extradental findings were displayed on the radiographs. Based on the results that some incidental findings were detected by the majority of participants (e.g. displaced tooth, incisor fracture), while others were detected by only a few examiners (e.g. apical radiolucency UJ incisor, arteriosclerosis of the external carotid artery), it can be stated that the detection of incidental findings in orthodontic X‑ray diagnostics varies in difficulty.

Overall, the participants detected only 39.1% of the incidental findings. This initially low detection rate can be explained by the phenomenon of “satisfaction of search”: Once an observer has detected a finding, he may stop looking for further findings and overlook them. This phenomenon is known in general radiology and has been investigated in numerous studies [4, 8, 13, 31]. Satisfaction of search has already been demonstrated in dentistry as well [17]. In addition, the participants detected significantly more incidental findings on OPTs than on LCs. One reason for this might be that the dental region is more clearly presented on OPTs. The regions depicted on LCs and OPTs, some of which are located far from the dental/jaw region (e.g. cranial base/cervical spine/soft tissue regions), might also cause incidental findings to be overlooked with higher frequency. This could also be supported by the fact that the investigators detected significantly more incidental dental than extradental findings. Currently, similar studies confirming this thesis cannot be found in the literature. It seems that orthodontists can reliably assess the radiographic structures within their own specialty, while their assessment routine outside the temporomandibular joint (TMJ) area seems to be deficient. The clinical relevance of these study results is highlighted by the fact that the finding of opacification in the area of the external carotid artery was associated with arteriosclerosis of the external carotid artery by less than 8% of the participants in the OPT of the adult patient in this study. Almog et al. [3] showed that OPTs are suitable as a screening tool for calcifications of the external carotid artery, even if further examinations have to be performed [6]. Early treatment of a circulatory disturbance can be a matter of life and death. This means that early detection of arteriosclerotic changes in the area of the external carotid artery is extremely important and therefore must never be overlooked as an incidental finding on a dental radiograph [2].

It was noticeable that significantly fewer incidental findings were detected on adult patient’s (LC 2 and OPT 2) radiographs than on those of adolescents (LC 1 and OPT 1). In contrast, combined LC and OPT surveys demonstrated that significantly more incidental findings were found on both radiographs with increasing patient age [16, 21]. Against the background of increasing numbers of adult patients attending orthodontic practices [24, 38], orthodontists should focus on the assessment of radiographs of this patient cohort, taking into account incidental findings. The factor of time for the assessment of the radiographs proved to be an essential aspect for a thorough and successful diagnosis of incidental findings in this study. With the results of the present study, important indications emerged that a more time-consuming diagnostic procedure was also associated with a higher number of detected findings, which is why a more generous time calculation may be recommended for this purpose in everyday practice. Especially against the background that falling behind a schedule, trying to keep to a schedule and constant time pressures are the main stressors in daily dental/medical patient care [5, 30], a short time contingent can have a negative impact on the quality of service [10, 28]. The results of this study highlight that despite the intense time pressure, radiographic assessment time should not be compromised. Sokolovskaya et al. [34] showed that among radiologists who halved their assessment time, the error rate increased from 10% to more than 26%. This study also concluded that the quality of the assessment depends on the assessment time and therefore a certain amount of time must be set aside for a detailed assessment. An analysis of professional experience and its influence on the number of incidental findings that were detected demonstrated that more incidental findings were identified with increasing professional experience. This coincides with the results of Geibel et al. [14]. With increasing professional experience, significantly more dental incidental findings were detected in this study. On the other hand, Bengtson et al. investigated the influence of professional experience on clinical circumstances, but could not confirm that dentists with more professional experience detected caries more reliably [7].

## Conclusion

The results of the present study clearly reveal that time is of outstanding importance for the practitioner in assessing orthodontic radiographs. In this context, the fact that many orthodontists perceive time constraints as the main stress factor should be considered critical. Even in routine clinical practice, special attention must be paid to a thorough and timely appropriate assessment of all regions on diagnostic radiographs. The factors time and professional experience can prevent the practitioner from overlooking findings that lie outside the orthodontic diagnostic focus. Furthermore, continuing medical education in orthodontics should address the assessment of orthodontic radiographs outside the orthodontic focus and increase interdisciplinary cooperation with other disciplines, such as radiology. Finally, considering the results of the presented study, periodic refresher courses in diagnostic imaging appear to be appropriate.
